# Health-Related Quality of Life 10 Years after Liver Transplantation: A Longitudinal Retrospective Review

**DOI:** 10.3390/diagnostics11010111

**Published:** 2021-01-12

**Authors:** Amber Hager, Diana Mager, Cheri Robert, David Nicholas, Susan Gilmour

**Affiliations:** 1 Department of Agricultural, Food & Nutritional Sciences, University of Alberta, Edmonton, AB T6G 2E1, Canada; ahager@ualberta.ca (A.H.); mager@ualberta.ca (D.M.); 2 Department of Pediatrics, University of Alberta, Edmonton, AB T6G 1C9, Canada; Cheri.Robert@albertahealthservices.ca; 3 Department of Social Work, University of Calgary, Calgary, AB T5J 4P6, Canada; nicholas@ucalgary.ca; 4 Division of Pediatric Gastroenterology & Nutrition/Transplant Services, The Stollery Children’s Hospital, Alberta Health Services, Edmonton, AB T6G 1C9, Canada

**Keywords:** health-related quality of life, pediatrics, children, liver transplantation, liver disease, liver, cholangiopathy

## Abstract

As survival post-liver transplantation (LTx) improves, it becomes increasingly important to understand how long-term health-related quality of life (HRQOL) is impacted. This was a longitudinal review examining HRQOL measured by Pediatric Liver Transplant Quality of Life (PeLTQL) in children between 8-17 years who underwent LTx (1.4 [0.8–3.3] years) at least one year prior to assessment. Demographic, medical, anthropometric, and HRQOL data (self-reported and parent proxy) were retrospectively collected over four years (2014–2017) at annual LTx clinic visits. The study included 35 patients (18M, 17F) and their parents/guardians. Parent-proxy and child PeLTQL scores (total, subdomain) showed good to excellent agreement (*p* > 0.05) and did not change over four years (*p* > 0.05). Younger age (<12 years) and Caucasian ancestry were associated with higher parental and self-reported perceptions of HRQOL, respectively (future health, coping and adjustment, total scores). Parent perceived lower HRQOL in social–emotional sub-domain (*p* = 0.03) and the child reported lower sub-domain scores related to coping and adjustment (*p* = 0.04) when the child was noted to have co-morbid conditions related to mental health and neurocognitive development (25.7%). While child–parent perceptions of HRQOL in a multi-ethnic population of pediatric LTx recipients remain unchanged 10 years post-LTx, adolescents of non-Caucasian ancestry remain a population at risk for lower HRQOL.

## 1. Introduction

Liver transplantation (LTx) is a life-saving procedure for children with end-stage liver disease (ESLD). Improvements in immunosuppression (IS) therapy, as well as other technological, medical, and surgical advances, have all contributed to improved outcomes such as graft and patient survival [1]. As survival increases, understanding factors affecting health-related quality of life (HRQOL) becomes a significant priority. HRQOL is a multidimensional construct encompassing a person’s physical and psychological health, personal beliefs, social relationships, and their relationship to their environment [2]. In children who have undergone LTx, HRQOL has shown to be significantly reduced compared to the healthy population [3,4,5]. Common issues affecting pediatric patients post-LTx are the need for daily immunosuppressant medication, long-term medical follow-up, risk of de novo malignancies, anxiety over liver rejection, and risk for depression [6,7,8].

Much of the literature has focused on generic measurements of HRQOL using non-specific measurement tools. While these tools are beneficial to compare HRQOL across a variety of populations, they are limited in addressing specific health issues associated with particular diseases or treatments [9]. More recently, a disease-specific tool for pediatric LTx patients has been developed (Pediatric Liver Transplant Quality of Life (PeLTQL)) [10]. To date, only two studies have used the PeLTQL questionnaire to describe HRQOL in LTx patients [11,12]. Furthermore, the majority of studies examining HRQOL in pediatric LTx populations have been cross-sectional analyses with only three longitudinal. The first for multiple solid organ transplant recipients [13], the second pre- and post-LTx [14], and a third recent study examining general HRQOL and cognitive functioning post-LTx [15]. However, there is still little known regarding longitudinal- and transplant-specific changes in HRQOL that may be experienced by the child post-LTx.

The objective of this study is to describe longitudinal changes in HRQOL in pediatric LTx recipients using a disease-specific tool. It was hypothesized that child/parent perceptions of HRQOL would significantly improve over time in children post-LTx.

## 2. Materials and Methods

This retrospective review collected demographic, medical, anthropometric, and HRQOL data annually (2014–2017) at routine visits at the Pediatric Liver Transplant Clinics at the Stollery Children’s Hospital in Edmonton, Canada. This review was approved by the Health Research Ethics Board at the University of Alberta (File Number: PRO00060677, 21 June 2016). HRQOL data were obtained from PeLTQL surveys [10] from children between the ages of 8–17 years who underwent LTx at our center at least one year prior to PeLTOL administration. Exclusion criteria included patients less than one-year post-LTx, patients younger than 8 years of age, and patients unable or unwilling to answer the HRQOL questionnaire. Both parent-proxy and child responses were collected.

Variables collected from electronic health records included socio-demographic (age, sex, ethnicity/race, grade level), liver disease diagnosis, pediatric end-stage liver disease score (PELD) or Model for End-Stage Liver Disease Score (MELD), immunosuppressive therapies, anthropometrics (weight, weight-z, height, height-z), serum aspartate transaminase (AST), alanine transaminase (ALT), gamma-glutamyltransferase (ϒ-GT), albumin, prothrombin time (PT), international normalized ratio (INR), total bilirubin, urea, creatinine, presence of co-morbid conditions (mental health, neurocognitive-developmental delay/psychiatric disorders, obesity), and access to rehabilitation and specialized school services.

The PeLTQL is self-administered and has both child and parent-proxy versions at a Flesch-Kincaid grade 5 reading level. The questionnaire consists of 26 questions across three domains (i.e., Future Health, Coping and Adjustment, and Social-Emotional) and requires five minutes to complete. The questions address concepts such as anxiety related to graft function/multi-organ function, abdominal scarring, etc. The raw scoring is based on a 5-point Likert scale, with a higher score indicating a better quality of life. These scores are then transformed and summed into a total final score from 0–100. Total scores and domains were calculated, and the summed score was divided by the number of items answered [10].

Data analysis was completed using the SAS 9.0 statistical software (SAS, Version 9.4; SAS Institute Inc, Cary, NC, USA). Data were expressed as mean ± SD or median and interquartile range (IQR), unless otherwise specified. The Shapiro–Wilk test was conducted to assess the normality of distribution. Nonparametric data were analyzed using Mann–Whitney. Repeated measures analysis of variance was performed to assess for differences in HRQOL (total scores and subdomain scores) over time. Univariate and multivariate analyses were conducted to assess potential relationships between primary outcomes of interest (HRQOL score and clinical outcomes). To assess for the potential effects of age, sex, and/or ethnicity, we treated these variables as categorical or class variables (12 years > and ≤12 years and ethnicity (Caucasian/non-Caucasian)) and used *t*-tests (normally distributed) and/or Mann–Whitney tests (non-normally distributed variables) to evaluate differences in total/subdomain scores of HRQOL. Agreement between HRQOL scores was evaluated using intraclass correlation coefficient (ICC), where ICC < 0.50, 0.50–0.75, > 0.75–0.90, and > 0.90 were indicative of poor, moderate, good, and excellent agreement, respectively [16]. A *p*-value < 0.05 was indicative of statistical significance.

## 3. Results

### 3.1. Demographics

Table 1 shows demographic and medical variables. A total of 35 eligible patients (18M, 17F) and their parents/guardians comprised the study cohort. In total, 79 questionnaires were completed over the four-year period. Median age at transplant was 1.4 years, IQR (0.8–3.3). Median age at primary assessment was 11.6 years, IQR (10.0–13.0). The median number of study visits was 2.0 years, IQR (2.0–3.0). The children had a median time from transplant to study assessment of 10.0 years, IQR (7.6–12.3). Out of available data, 71% were of Caucasian ethnicity. The majority of patients were transplanted for biliary atresia (54.3%). Other common diagnoses included fulminant liver failure (5.7%), primary sclerosing cholangitis (8.6%), metabolic liver diseases (8.6%) and other (22.9%). Twelve patients (34.3%) experienced previous acute/chronic rejection (>6 months from PeLTQL administration). No participants experienced acute/chronic rejection within the past six months of PeLTQL administration.

### 3.2. Health-Related Quality of Life

**Figure 1:**
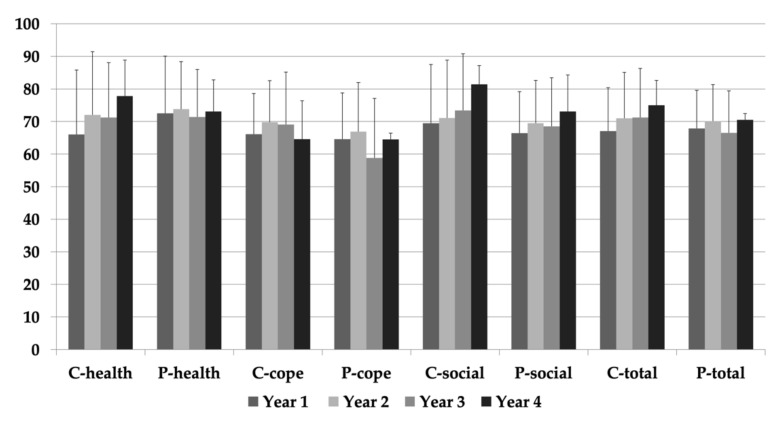
Total and sub-domain scores (Future Health [health], Coping and Adjustment [cope] and Social-Emotional [social]) for both child (C) and parental (P) health-related quality of life (HRQOL) reports over four years. All values expressed as mean ± standard error. No statistical significance noted.

Sub-domain (future health, coping and adjustment, social-emotional) and total scores over the four years for both child and parent-proxy are presented in Figure 1. Parent-proxy and child PeLTQL scores showed good-excellent agreement in sub-domain and total scores (*p* > 0.05). Parent-proxy and child PeLTQL scores (total, composite) did not change over four years (*p* > 0.05).

### 3.3. Ethnicity and Age

**Figure 2:**
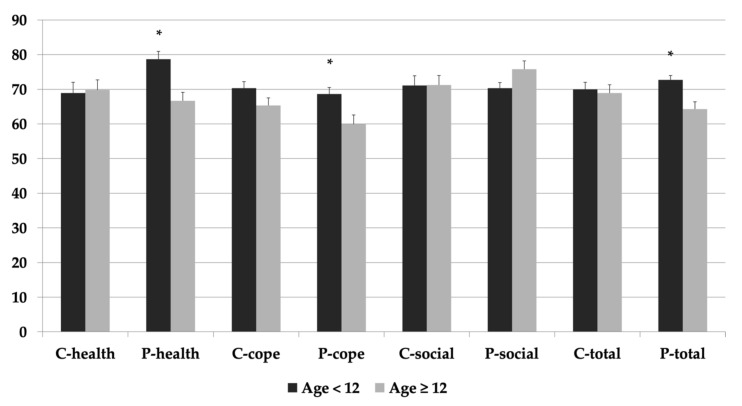
Total and sub-domain scores (Future Health [health], Coping and Adjustment [cope] and Social-Emotional [social]) for both child (C) and parental (P) HRQOL reports for children ≥12 and <12 years of age. All values expressed as mean ± standard error. * Significant at *p* < 0.05.

**Figure 3:**
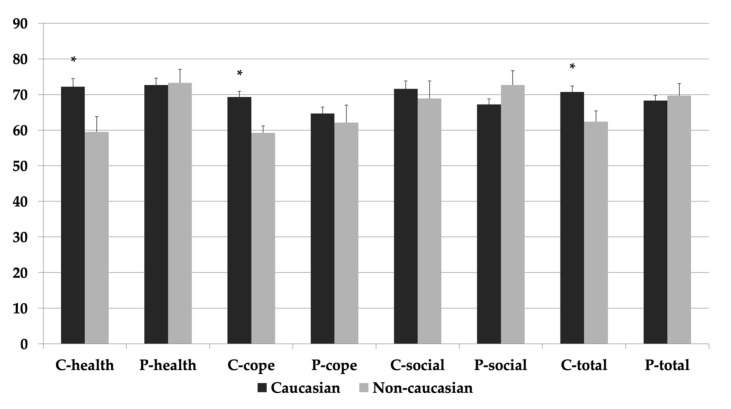
Total and sub-domain scores (Future Health [health], Coping and Adjustment [cope] and Social-Emotional [social]) for both child (C) and parental (P) HRQOL reports for children of Caucasian and non-Caucasian ancestry. All values expressed as mean ± standard error. * Significant at *p* < 0.05.

Younger age of the child (< 12 years) was associated with higher parental perceptions of HRQOL for future health and coping and adjustment subdomains, as well as total scores when compared to older children (≥ 12 years) (*p* < 0.05) (Figure 2). Caucasian ethnicity was associated with higher child perceptions of HRQOL scores for future health and coping and adjustment subdomains, as well as total scores when compared to children of non-Caucasian ancestry (*p* < 0.05) (Figure 3). These relationships were not impacted after adjustment for the potential confounding effects of age at survey, age at transplantation, sex, and anthropometric variables (*p* > 0.05)

### 3.4. Medical Factors and Co-Morbidities

Parent perceived lower HRQOL in social sub-domains (*p* = 0.03) and the child reported lower sub-domain scores related to coping (*p* = 0.04) when the child was noted to have co-morbid conditions related to mental health and neurocognitive development. Neurocognitive/mental health issues were identified in approximately 25.7% of the participants in this review. There was little incidence of access to rehab services (*n* = 1, 2.9%), specialized school programming (*n* = 1, 2.9%), and guardianship orders (*n* = 1, 2.9%). No other significant associations between total/sub-domain scores (parent-proxy/child report) for PeLTQL and liver disease diagnosis, immunosuppression (type/dose), cumulative number of episode of acute or chronic rejection, laboratory biochemistries (AST, ALT, ϒGT, total bilirubin, albumin, PT, INR), PELD/MELD, sex, access to rehabilitation services, and anthropometric variables were found (*p* > 0.05).

## 4. Discussion

This study longitudinally evaluated parental and child HRQOL over four years in children who underwent LTx using a validated tool (PeLTQL) that assesses important constructs related to HRQOL. HRQOL is an important concept to address post-LTx, as many children experience challenges associated with the ability to perform routine activities of daily life such as participation in school, recreational activities, and sports [17,18]. Previous research from the Studies of Pediatric Liver Transplant (SPLIT) consortium across the world has examined HRQOL in pediatric LTx using generic questionnaires [3,15,19,20,21]. From their findings, it is known that HRQOL is consistently reduced when compared to a healthy population. This study is unique in that we are one of the few clinics to routinely measure HRQOL using a tool that focuses on the specific HRQOL considerations related to LTx. In this study, HRQOL was stable over four years (total, future health, coping and adjustment, social-emotional subdomains) with good to excellent agreement between parental and child perceptions. Younger children (< 12 years) and children of Caucasian ancestry were found to have higher parental and child perceptions of HRQOL, respectively.

The stability of HRQOL over the four years is consistent with a recently published longitudinal study examining HRQOL in pediatric LTx patients (from 8 to 16 years) using a generic assessment tool (PedsQL 4.0 [22]) [15]. Ohnemus et al. found that even though HRQOL was stable over the study period, parent-reported HRQOL was significantly lower in all domains when compared to the healthy reference population [15]. Additionally, they found that child self-reported HRQOL was significantly lower in social and school functioning domains when compared to the reference population. No significant differences were noted between demographic (age, sex, ethnicity) or medical variables (liver disease). Similarly, other studies have found that even ten years post-LTx, child and parent-proxy perceptions of HRQOL were significantly lower when compared with healthy children [23]. These findings suggest that although HRQOL remains unchanged, both parent and child perceptions of HRQOL remain significantly reduced when compared to that of healthy children. A recent qualitative study from the perspective of health-care providers noted that a patient’s ability to cope and adapt after LTx was most notable in children who had a LTx at a younger age [7]. Therefore, the stability seen over four years may also be explained by the relatively early age at time of LTx (1.4 years) of our population.

Our data may be compared to a cross-sectional study using PeLTQL, which found median total parent/child scores to be 78.6/77.1, respectively [12]. This is higher than our total scores of 70.3/70.2. This difference could be explained by the younger age (8.9 years) of their population compared to ours (12.3 years). After correcting for age, our data produced comparable results of median total parent/child scores to be 77.9/77.9, respectively. Across LTx and other disease populations, HRQOL has been shown to be worse in adolescents [24,25]. Likewise, our finding that younger age (< 12 years) is associated with higher perceptions of HRQOL (total score and coping sub-domains) is consistent with this literature. However, it is possible that as a child ages, older age (> 12 years) may be associated with worse perceptions of HRQOL as adolescents may struggle with body image, disease self-management, and medication adherence as a teenager [7] as well as fear of participation in sports or other activities [17,18].

This study found that Caucasian ethnicity was associated with higher child-reported HRQOL scores (future health, coping and adjustment, total) when compared to that of non-Caucasian ancestry. Associations between HRQOL and ethnicity are inconsistent in the literature. A study by Bucuvalas et al. similarly found that Caucasian children had higher perceptions of physical functioning when compared to non-Caucasian ethnicity [4]. However, multiple studies either did not examine ethnicity in relation to HRQOL or did not find any significant associations [3,5,11,12,13,15,20,23]. In adults, ethnicity has been shown to impact all stages of the transplant trajectory from longer times on the waitlist [26], increased waitlist mortality to short- and long-term patient and graft survival post-LTx [27]. In pediatrics, a retrospective review found similar existence of ethnic disparities in LTx patients with graft and patient survival higher for Caucasian vs. non-Caucasian minorities [28]. These differences are likely attributed to a variety of social variables. For example, differences may be related to access and utilization of health care, perhaps due to language barriers or different perceptions surrounding health. Additionally, differences in socioeconomic status may affect the ability to pay for certain medical services, medications, and transit to doctor’s appointments. Lastly, bias and racial discrimination within the healthcare system may lead to poorer self-rated health. Together, all of these factors could contribute to decreased perceptions of HRQOL of life.

Although simpler IS regimens have been associated with better HRQOL [12], we did not find any associations. Differences may have been due to an increased percentage of 91% (*n* = 45/49) of our population on IS monotherapy in comparison to that study (64%). Another reason for the difference could be related to the fact that none of our patients were on steroid therapy. Steroid therapy has been shown to be associated with poorer quality of life in transplant patients due to effects of steroid withdrawal as well as more long-term side effects that can affect HRQOL such as reduced growth and low bone mineral content [29]. Therefore, the fact that most of our cohort was on tacrolimus monotherapy and on steroid-free IS regimens throughout the study period could be a reason we did not see any differences in HRQOL. Tacrolimus has common side effects such as nephrotoxicity, neurotoxicity, cardiovascular disease, and diabetes [30]. Although none of these side effects were noted in our population, it is still important to recognize the short- and long-term side effects of IS therapy.

Similarly, although rejection episodes have been associated with HRQOL [13], this study found no associations. This is likely explained by the fact none of our patients were experiencing active rejection within the past six months of PeLTQL administration.

A strength of this study was the use of a disease-specific tool (PeLTQL) for measuring HRQOL. By using a liver disease-focused tool, we were able to capture transplant relevant HRQOL issues facing children post-LTx. The use of PeLTQL has been validated against the generic PedsQL 4.0 tool and has shown a strong positive correlation and similar HRQOL properties [10]. Therefore, although we were not able to compare our results directly to a healthy population, the PeLTQL is still able to capture similar general properties. This study has some other limitations including the smaller sample size and single transplant center.

In summary, this study demonstrates that while child–parent perceptions of HRQOL in a multi-ethnic population of pediatric LTx recipients remains unchanged 10 years post-LTx, adolescents of non-Caucasian ancestry remain a population at risk for lower HRQOL. This study highlights the need to provide both medical and psychosocial support to adolescents and young adults, particularly of ethnic minorities, as they transition into adult care and self-management of disease. Further longitudinal multicenter prospective studies using both generic and disease-specific tools are warranted to fully understand the dynamic factors affecting long-term HRQOL and, therefore, the steps clinicians and families can take to improve the HRQOL of the child.

## Figures and Tables

**Table 1:** Demographic and medical variables.

	*n*	%	Median (IQR)
**Patient Characteristics at LTx**			
**Sex**			
Male	18	51.4	
Female	17	48.6	
Age at LTx, yrs	35		1.4 (0.8–3.3)
**Liver Disease Score**			
PELD	34	97.1	15.4 (7.0–22.8)
MELD	1	2.9	17.0
**Anthropometrics**			
Weight-z at LTx	29		−0.4 (−0.8–0.6)
Height -z at LTx	21		−0.9 (−1.4–0.6)
**Graft Type**			
Living	16	45.7	
Cadaveric	19	54.3	
**Indication for Transplant**			
Biliary Atresia	19	54.3	
Fulminant Liver Failure	2	5.7	
Metabolic Liver Diseases	3	8.6	
PSC	3	8.6	
Other	8	22.9	
**Race**			
Caucasian	12	34.3	
Aboriginal	3	8.6	
Asian	1	2.9	
Black	1	2.9	
Unknown	18	51.4	
**Patient Characteristics at PeLTQL**			
Age at initial PeLTQL, yrs	35		11.6 (10.0–13.0)
Age across all PeLTQL, yrs	79		12.3 (10.8–13.8)
< 12	36	45.6	10.5 (9.6–11.4)
≥ 12	48	54.4	13.9 (13.0–14.8)
**Anthropometrics at Initial PeLTQL**			
Weight-z	29		−0.2 (−0.4–0.6)
Height-z	29		0.5 (−0.50–0.8)
Interval from LTx to all PeLTQL	79		10.0 (7.6–12.3)
Interval from LTx to initial PeLTQL	35		8.8 (7.0–10.9)
**Medications**			
Number of medications	49		
Tacrolimus Monotherapy ^†^	45	91.8	1.0 (1.0–2.0)
IS Polytherapy ^†,^*	4	8.2	
No medication data available	30		
**Past Rejection (> 6mo)**	12	34.3	
Acute	8	22.9	
Chronic	4	11.4	

Immunosuppression (IS); Liver Transplantation (LTx); Model for End-Stage Liver Disease Score (MELD); Pediatric End-Stage Liver Disease Score (PELD); Pediatric Liver Transplant Quality of Life (PeLTQL); Primary Sclerosing Cholangitis (PSC). ^†^ Percentage out of 49 available medications lists. * Polytherapy included Tacrolimus and Mycophenolate Mofetil.

## Data Availability

Data is contained within the article.
